# The endless visuomotor calibration of reach-to-grasp actions

**DOI:** 10.1038/s41598-018-33009-6

**Published:** 2018-10-04

**Authors:** Robert Volcic, Fulvio Domini

**Affiliations:** 1 Department of Psychology, New York University Abu Dhabi, PO Box 129188, Abu Dhabi, United Arab Emirates; 2 Center for Neuroscience and Cognitive Systems@UniTn, Istituto Italiano di Tecnologia, 38068 Rovereto, Italy; 3 Department of Cognitive, Linguistic and Psychological Sciences, Brown University, Providence, RI 02912 USA

## Abstract

It is reasonable to assume that when we grasp an object we carry out the movement based only on the currently available sensory information. Unfortunately, our senses are often prone to err. Here, we show that the visuomotor system exploits the mismatch between the predicted and sensory outcomes of the immediately preceding action (sensory prediction error) to attain a degree of robustness against the fallibility of our perceptual processes. Participants performed reach-to-grasp movements toward objects presented at eye level at various distances. Grip aperture was affected by the object distance, even though both visual feedback of the hand and haptic feedback were provided. Crucially, grip aperture as well as the trajectory of the hand were systematically influenced also by the immediately preceding action. These results are well predicted by a model that modifies an internal state of the visuomotor system by adjusting the visuomotor mapping based on the sensory prediction errors. In sum, the visuomotor system appears to be in a constant fine-tuning process which makes the generation and control of grasping movements more resistant to interferences caused by our perceptual errors.

## Introduction

The act of reaching for an object and grasping it is the result of a complex chain of intermediate processes that transform the retinal input into a desired motor goal. The problem of vision for action is to understand how visual information is encoded before it is mapped to the appropriate movement execution. A vastly influential theory proposes that to perform this common action there is a dedicated visual system that computes “the required coordinates for action at the very moment the movements are performed” in order to “reflect the real metrics of the world”^1^. As a consequence, it is to expect that in normal circumstances previous actions have little or no bearing on the current one, since the information immediately available is trustworthy and sufficient to fulfill the desired goal. In other words, metric accuracy makes any refinement or calibration of the motor mapping, on the basis of preceding instances of normal grasping movements, unnecessary.

**Figure 1:**
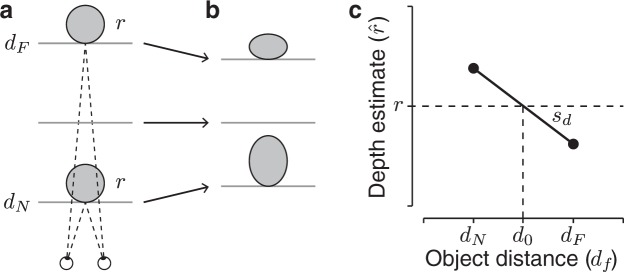
Shape from stereopsis. (**a**) Two objects of the same size *r* are placed at two distances (*d*_*N*_ and *d*_*F*_). (**b**) Due to wrong encoding of distance from ocular vergence, the close object is perceived farther and deeper and the far object is perceived closer and shallower. (**b**) A linear function of negative slope (*s*_*d*_) that represents the relationship between depth estimates and object distance.

However, evidence against metric accuracy has been shown in previous studies, where participants grasped objects whose location and 3D structure were only specified by ocular vergence and retinal disparities^2–6^. Interestingly, grasping actions as well as perceptual judgments seem to rely on the same biased depth estimates, which systematically depend on the object distance^7–12^. Objects close to the observer are perceived farther and deeper than they actually are, while objects far from the observer are perceived closer and shallower than they are (Fig. 1a,b). These distortions can be explained with the wrong encoding of distance information from ocular vergence and can be modeled, in first approximation, with a linear function of negative slope relating depth estimates to object distance (Fig. 1c)^11^. Thus, each time a grasping movement ends, there is a resulting conflict between the felt depth of the object, which is constant, with a decreasing visually perceived depth.

In this study, we will provide evidence against the idea that actions are temporally-isolated, that is, that they rely only on current estimates. We hypothesize that the failure to estimate veridical metric properties of an object leads to an inaccurate movement plan, which leads to an error and, subsequently, an update to the visuomotor mapping. We thus hypothesize that (1) a sensory prediction error is generated after every grasp; (2) the detected error yields a correction of the planned grip aperture.

We tested this idea in an experimental environment where participants repeatedly grasped the same object. The object appeared as a computer generated random-dot sphere presented at eye level at various distances in the sagittal plane in an otherwise dark visual-field. Importantly, participants received haptic feedback upon termination of the grasp; thus, signaling the correct size and position of the object at each trial. To ensure that biases did not accumulate over time, we counterbalanced the trials using de Bruijn sequences^13^ (see Stimulus ordering section). The required grasp necessitated the visual estimate of the object depth, which was solely conveyed by binocular information. If this information were sufficient for an accurate assessment of the object structure, the retinal disparities should be scaled by the object distance detected via vergence information. Instead, we show that when reach-to-grasp requires depth estimates from binocular disparities (1) the visual system does not gain access to “truthful” physical information, and, (2) the visuomotor mapping is computed not only on the basis of current visual information but also on previous actions.

The experiments reported here were designed as a direct test of the hypothesis that grasping actions are based on a combination of the current visual information and the outcome of the immediately preceding action. To vary the magnitude of sensory-prediction errors, we manipulated the viewing geometry. In Experiment 1, participants grasped the object along the depth axis and thus the digits and the final contact points were separated mainly in the depth dimension. In Experiment 2, participants grasped the object along an oblique axis and thus the digits and the final contact points were separated in both the depth and the frontoparallel dimensions. To quantify the effect that the outcome of the immediately preceding action has on grasping, we measured the effect of the current and previous object distance on the grip aperture and on the hand position.

## Results

### Predictions

Previous work has found that perceived depth decreases with distance. We propose that the motor system tries to overcome these biased estimates by the trial-by-trial adjustment of a visuomotor mapping based on sensory-prediction errors. Here, we formalize this connection by developing a model that modifies an internal state of the visuomotor system. This framework has been used extensively in order to test how accurate movement coordination is maintained following visual perturbations, showing that humans can rapidly adjust visuomotor mappings based on feedback about movement outcomes^14–17^ via sensory-prediction errors^18^. In order to assess the generality of the model predictions we tested participants in two kinds of grasps relying on size estimates which were influenced by distance information to a different degree. In one condition, participants were grasping objects along the depth dimension, that is, forcing the thumb and index finger to land at the closest and farthest surface points of a sphere. This front-to-back grasp required the estimate of the object depth $\hat{z}$, which is subject to the systematic biases described above. In another condition, the sphere was grasped with an oblique grasp. The magnitude of this grasp depended equally on depth information and on 2D size information. It was therefore subject to a lesser degree to biases caused by lack of depth constancy. Thus, in both conditions, the grasp relied on a size estimate $\hat{r}$, which in first approximation was a linear function of object distance *d*_*f*_: $\hat{r}$ = *r* + *s*_*d*_(*d*_*f*_ − *d*_0_), where *r* is the true size and *d*_0_ the distance at which size estimates are veridical (see Fig. 1).

Suppose that the planned grasp at the trial (*n* − 1) is based on a size estimate ${\hat{r}}_{n-1}$ and on an internal state *X*_*n*−1_: ${r}_{{p}_{n-1}}={\hat{r}}_{n-1}+{X}_{n-1}$. The difference between this quantity and the actual size *r*, conveyed by haptic feedback, is what we define as the *sensory prediction error*, ${\varepsilon }_{{g}_{n-1}}={r}_{{p}_{n-1}}-r$. Note that this error is not what we claim to be actually measured by the visuomotor system. We simply assume that this quantity is linearly related to the discrepancy between the expected kinematics of the movement and those experienced through proprioception^19–21^. For example, if the planned grasp is too large (${r}_{{p}_{n-1}} > r$), the fingers will touch the object later than expected. The detected error will then produce a modification of the following grasp through a change in the internal state: ${X}_{n}=a{X}_{n-1}-b{\varepsilon }_{{g}_{n-1}}$, where *a* is a retention parameter and *b* a correction parameter.

This model allows the formulation of precise quantitative predictions of the effect of the previous trial on the current trial grasp (see Modeling section for more details). Let us indicate with ${g}_{{r}_{p}}(t)$ the instantaneous rate of change of the grip aperture^22^ with the planned grasp magnitude *r*_*p*_. It can be shown that at a time instant *t* the slopes of the functions relating the grip aperture to (1) the distance of the object on the current trial *n*, and, (2) the distance of the object on the previous trial (*n* − 1), are proportional to each other (with opposite sign):

1 $${{\rm{slope}}}_{n}(t)={g}_{{r}_{p}}(t){s}_{d}$$

2 $${{\rm{slope}}}_{n-1}(t)=-\,b{g}_{{r}_{p}}(t){s}_{d}.$$

From here on, we will denote these slopes as the current distance effect and the previous distance effect, respectively. The slope_*n*_(*t*) and the slope_*n*−1_(*t*) are time functions which allow the formulation of specific predictions. First, slope_*n*_(*t*) is a function which should maintain a negative value throughout the movement. Second, slope_*n*−1_(*t*) should be equal to slope_*n*_(*t*), but attenuated by the correcting factor *b* and with opposite sign. Third, assuming a small variability of the correction parameter *b* across individuals the previous equations predict that the magnitude of the previous trial effect increases with the magnitude of the current trial effect. That is, participants who exhibit sizable lack of depth constancy in their grasps will also show robust corrections and therefore strong effect of previous trial. On the other hand, other individuals will reveal mild effects of both previous and current distance. Fourth, since for an oblique grasp *s*_*d*_ is smaller than for a grasp in depth the amplitude of both functions will be smaller as well.

As mentioned before, the lack of depth constancy results from inaccurate estimates of object distance leading to incorrect scaling of binocular disparities. The estimated distance $\hat{d}$ of an object is related to the actual distance *d* through a linear function of slope *k* < 1. If this function hits veridicality when the object is located at a distance *d*_0_ then objects closer than *d*_0_ will appear farther than they are and objects farther than *d*_0_ will appear closer than they are. If during grasping the reaching component is subject to these biases then it can be shown that the instantaneous slope relating hand position to current and previous object distance is given by:

3 $${{\rm{slope}}}_{hn}(t)={h}_{{d}_{p}}(t)k$$

4 $${{\rm{slope}}}_{hn-1}(t)=-b{h}_{{d}_{p}}(t)(k-\mathrm{1)}$$

Unlike the equivalent descriptions relative to the grip apertures these slopes are not proportional to each other, making it therefore problematic for hand position to predict the previous trial effect from the current trial effect. However, it is possible to predict the sign of the previous trial effect. Since *k* < 1 and *b* and ${h}_{{d}_{p}}(t)$ are positive, this slope will be positive as well.

### Behavioral results

**Figure 2:**
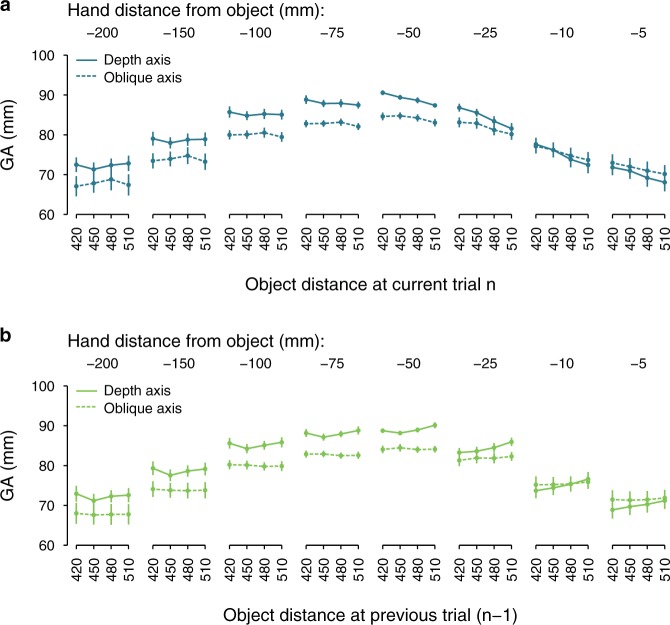
Distance effects on grip aperture. (**a**) Grip aperture (GA) as a function of the object’s distance at the current trial (dark blue lines). (**b**) Grip aperture (GA) as a function of the object’s distance at the previous trial (light green lines). Grasping was performed along the object’s depth axis (Experiment 1, continuous lines) or along the object’s oblique axis (Experiment 2, dashed lines). Separate panels represent the grip aperture when the hand was at different points of the movement path (the more negative values correspond to points of the movement path that are further away from the object). Error bars represent the standard error of the mean.

Figure 2 shows the progress of the grip aperture over the last 200 mm of the movement. The grip aperture reached its peak when the hand was approximately 50 mm from the object and then the grip started to close before making contact with the object. The grip aperture was slightly larger when grasping was performed along the depth axis, which could be due to stronger repulsive effects of the object along this dimension^23,24^. However, the aspect of major interest here was the modulation of the grip aperture as a function of the current distance (Fig. 2a). Grip apertures were consistently larger when the grasping action was directed toward nearer objects and this pattern was, as expected, more pronounced when objects were grasped along the depth axis (continuous lines) than along the oblique axis (dashed lines). Thus, the effect the current distance has on the grip aperture shows that reach-to-grasp actions are not based on metric visual information.

If the visuomotor mapping is the result of not just the current visual information, but also of the preceding action, the grip apertures should depend on the distance at which the object was grasped at the previous trial. In particular, the previous distance effect should be to some degree weaker, but, most importantly, it should be opposite in sign to the current distance effect. Indeed, this is exactly what we have observed here (Fig. 2b). Grip apertures were larger when the preceding action was directed toward more distant objects.

**Figure 3:**
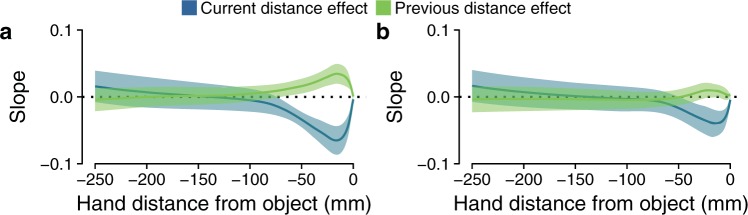
Current and previous distance effects on the grip aperture. Slope profiles representing the current distance effect (dark blue line) and the previous distance effect (light green line) on the grip aperture along the movement path when grasping along the depth axis (**a**) and along the oblique axis (**b**). The bands represent the 95% confidence interval of the slope parameters.

To fully characterize both the current and previous distance effects we created the slope profiles which represent the progress of these effects at each 1 mm step of the last part of the movement trajectory (for more details, see Data analysis section). The slope profiles of these effects on the grip aperture are shown in Fig. 3. The current distance effect (dark blue lines) arose toward the end of the movement and it reached a negative peak equal to −0.07 and −0.04 when grasping along the depth axis and along the oblique axis, respectively. These negative peaks are equivalent to a grip aperture difference of 6.3 mm and 3.6 mm between the closest (420 mm) and farthest (510 mm) positions of the object. Participants performed consistently larger grip apertures for objects closer to their body. The sign and magnitude of this effect closely mimics the effects found in previous studies. For instance, the slopes reported for a grasping^5^ and for a perceptual task^11^ were −0.05 and −0.03, respectively.

In contrast, the previous distance effect (light green lines) developed in the opposite direction, but in the same part of the movement, and it reached a positive peak equal to 0.03 and 0.01 when grasping along the depth axis and along the oblique axis, respectively. These positive peaks are equivalent to a grip aperture difference of 2.7 mm and 0.9 mm if the movement was preceded by a movement toward the closest or farthest position of the object. Participants thus opened their fingers more after having grasped an object at a far distance than after having grasped the same object at a closer distance. As the size of the object was kept constant throughout the experiment, it is needless to say that both the current and the previous distance effects dropped to zero the moment the fingers enclosed the object.

**Figure 4:**
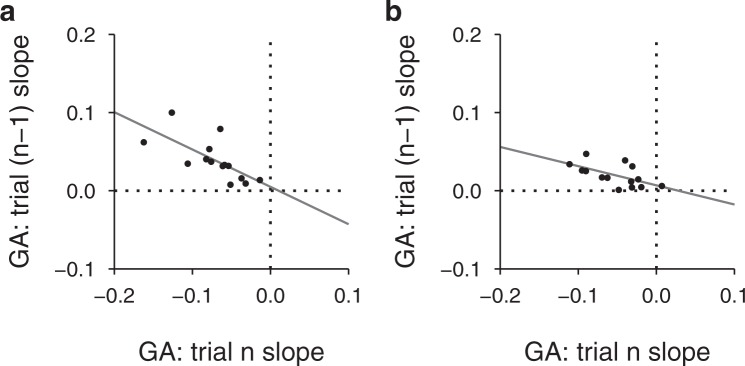
Relationship between individual current and previous distance effects on grip aperture. (**a**) Individual data from Experiment 1 (grasping along the object’s depth axis). (**b**) Individual data from Experiment 2 (grasping along the object’s oblique axis). Solid lines show the linear regression fits.

The relationship between the current and the previous distance effects on grip aperture was clearly visible also at the per-participant level (Fig. 4). The correlations between these effects were −0.698 (95% BCa CI: −0.872, −0.181) and −0.596 (95% BCa CI: −0.826, −0.137) when grasping along the depth axis and along the oblique axis, respectively. All participants showed a negative current distance effect and, conversely, all participants showed a positive previous distance effect. In both experiments, the previous distance effect was consistently smaller than the current distance effect as shown by the linear regression fits with the slopes being −0.478 (95% BCa CI: −0.912, −0.263) and −0.246 (95% BCa CI: −0.432, −0.127), and the intercepts being 0.005 (95% BCa CI: −0.018, 0.026) and 0.007 (95% BCa CI: −0.002, 0.019).

**Figure 5:**
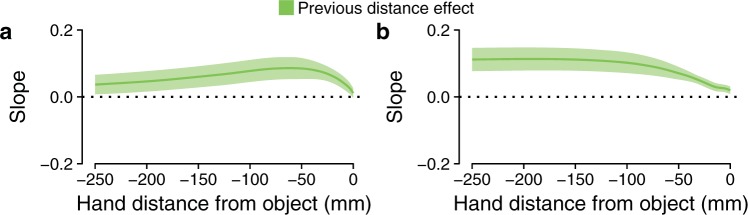
Previous distance effects on the hand position. Slope profiles representing the previous distance effect on the hand position along the movement path when grasping along the depth axis (**a**) and along the oblique axis (**b**). The bands represent the 95% confidence interval of the slope parameters.

Interestingly, not only was the grip aperture affected by the distance at which the object was presented at the preceding trial, but also the way the hand was transported. The slope profiles of the previous distance effect on the hand position are positive already when the hand was still 250 mm from the object (Fig. 5). This means that the hand was consistently displaced closer or further away (along the sagittal axis) after having performed the previous movement toward a closer or farther object. The positive peaks of these slope profiles were 0.09 and 0.11 when grasping along the depth axis and along the oblique axis, respectively. These values are equivalent to a hand position difference of 8.1 mm and 9.9 mm if the movement was preceded by a movement toward the closest or farthest position of the object.

## Discussion

Our study showed that reach-to-grasp actions are based not only on the current visual information, but also on the immediately preceding action. When grasping the exact same object, the grip aperture and the path of the hand were predictably modulated by the distance at which the object was located on the previous trial. Notably, these effects occurred despite the availability of on-line visual feedback and consistent haptic information, and despite the fact that successive actions were separated in time by several seconds.

The depth estimates from binocular disparities required to perform successful reach-to-grasp movements do not reflect the metric properties of the target object, but they instead systematically depend on the object distance, mirroring perception^2–6^. The grip aperture tends to decrease with the increase of object egocentric distance. Thus, the predicted and the sensory outcomes of the action do not generally match. However, this sensory prediction error (i.e., the mismatch between predicted and sensory outcomes) provides the necessary information to rectify the planning of the next grasping action and thus the next action will be systematically affected by the immediately preceding one. What might seem a maladaptive feature actually reveals the exceptional plasticity of the visuomotor system.

In particular, we have modeled the previous distance effect as a direct consequence of the sensory prediction error caused by the suboptimal depth estimate on which the grasping movement is based. Thus, according to the model (1) the previous distance effect was expected to be opposite in sign to the current distance effect, (2) the magnitude of the previous distance effect was expected to depend on the magnitude of the current distance effect, and, (3) the relationship between the magnitude of the current and previous distance effects should also hold at the per-participant level. Indeed, this is what we have observed. First, the larger the current object distance, the smaller the grip aperture was, and, the larger the previous object distance, the larger the grip aperture was. Second, the current distance effect and, consequently, the previous distance effect were stronger when participants grasped objects along the depth dimension, which required an estimate of the object depth only, than along an oblique orientation, which required an estimate that only partially depended on the object depth. Third, participants who exhibited larger current distance effects revealed consistently stronger previous distance effects. This relationship is thus in line with the idea that a sizable lack of depth constancy produces larger sensory prediction errors which in turn require larger corrections.

According to our model, the current and the previous distance effects should have been visible already earlier in the movement. One possible explanation for the later appearance of these effects is that, as the hand moves to grasp an object, the preshaping of the hand mainly occurs in the latter parts of the movement just before the hand encloses the object. Any differences in grip aperture prior to this last phase of the movement could thus be masked by the higher variability that generally characterizes the initial phases of the movement. However, the previous distance effect on the hand position was visible already much earlier, as predicted by the model.

How is it that participants never learn the correct visuomotor mapping even though the sensory prediction errors regularly provide feedback about how to improve the motor behavior? The reason is straightforward. The direction of the error varied from trial to trial and thus the corrections were made in either direction depending on where the objects were at the current and previous trials. If the grip aperture during a movement toward a distant object was not sufficiently large (i.e., safety margin smaller than expected), the grip aperture was increased on the subsequent movement. However, if the subsequent movement was directed toward a nearer object, the grip aperture would now likely be too large. Thus, on the next trial the grip aperture should be reduced again, and, so forth. It is worth noting that the veridical haptic feedback about the object size wasn’t by itself sufficient to establish the correct visuomotor mapping. The exact same reasoning can be applied to account for the failure in learning the optimal way to transport the hand toward the object. The visuomotor mapping was constantly updated with conflicting information and, therefore, the error corrections simply neutralized one another^19–21^.

The idea that a past event can influence a future event is certainly not new^25^. Sequential effects reported in the visuomotor literature are usually perseveration effects, that is, a movement tends to resemble that on the previous trial^26^. For instance, when participants were asked to make arm movements and they occasionally had to avoid an obstacle half-way between the start and the target, they made curved rather than straight trajectories when an obstacle-absent trial was preceded by an obstacle trial^27,28^. Or, the grip aperture tended to be smaller following a smaller object and larger following a larger object^29^. These findings are generally explained with a motor priming or sensorimotor memory account according to which motor plans (or sensorimotor memories) remain active even once the action is already executed and they are thus reused on ensuing movements, especially when the computational cost of planning a new movement outweighs the biomechanical cost of performing a suboptimal movement^27–35^. The sequential effects we observed in the present study were not simply due to perseveration; quite the contrary, they instead reflect the endless visuomotor calibration processes which promote the best possible behavior despite motor noise and the persistent fallibility of our perceptual processes.

## Methods

### Participants

Twenty-eight undergraduate students (16 females) with normal or corrected-to-normal vision participated in this study. All of the participants were naïve to the purpose of the study and received monetary compensation for their time and effort. Half of them participated in Experiment 1, the other half in Experiment 2. Written informed consent was obtained from all participants. Experimental procedures were approved by the ethics board of the University of Trento (Comitato Etico per la Sperimentazione con l′Essere Vivente) and were in accordance with national legislation and the Code of Ethical Principles for Medical Research Involving Human Subjects of the World Medical Association (Declaration of Helsinki).

### Apparatus

Participants were seated in front of a front-silvered 400 × 300 mm mirror which was slanted at 45° with respect to the sagittal body mid-line of the participant. The mirror reflected the image displayed on the 19″ CRT monitor (ViewSonic 9613) which was positioned to the left of the mirror. This configuration produced the illusion that the rendered 3D objects were displayed directly in front of the participant. The monitor was mounted on a linear positioning stage (Velmex Inc., Bloomfield, NY, USA) and its position was adjusted on each trial to equal the distance from the the virtual object to the eyes of the participant to provide consistent vergence and accommodative information. Visual stimuli were presented in 3D by using a a frame interlacing technique together with liquid crystal FE-1 goggles (Cambridge Research Systems, Cambridge, UK) synchronized to the monitor’s frame rate. For stimulus presentation and response recording we used a custom C++ program. The estimated lag of the system was 27.9 ± 1.3 ms^36^.

The Optotrak Certus motion capture system with two position sensors (Northern Digital Inc., Waterloo, Ontario, Canada) was used to acquire head, wrist, index and thumb on-line movements at a sampling rate of 100 Hz. Head movements were measured to update the participant’s viewpoint for a correct geometrical projection of the stimulus. The position and orientation of the head was tracked with three infrared-emitting diodes which were positioned on the back of the head. The position of the tip of each digit was calculated during the system calibration phase with respect to three infrared-emitting diodes attached on each distal phalanx. More details about this setup are available in Nicolini *et al*.^37^.

**Figure 6:**
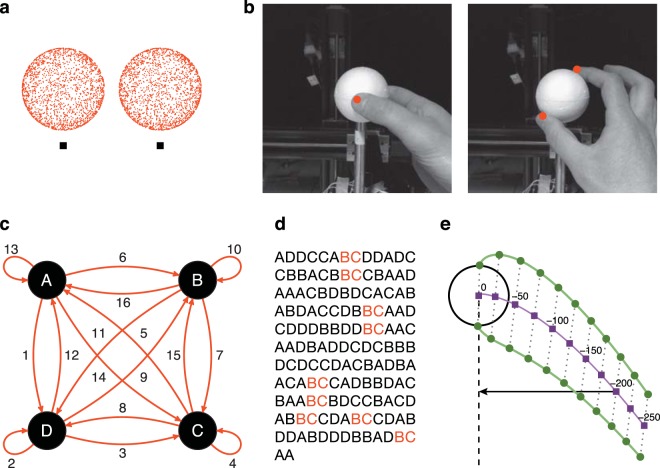
Methods. (**a**) The three-dimensional structure of the stimulus can be observed by cross-fusing the two images stereoscopically. (**b**) Grasping along the depth axis (left image) and along the oblique axis (right image). Participants never had full vision of the hand, only visual feedback about the fingertips was provided (red dots). (**c**) A de Bruijn graph for *n* = 2 and a four-character alphabet composed of the characters A, B, C, and D which represent the four object’s distances. Following the numbered edges in order from 1 to 16 traces an Eulerian cycle AD, DD, DC, CC, CA, AB, BC, CD, DB, BB, BD, DA, AA, AC, CB, BA. By appending the first element to the tail and by taking the first character of each of these elements we obtain the de Bruijn sequence ADDCCABCDBBDAACBA without repetitions. (**d**) An example of a 145-long de Bruijn sequence with the repetition factor set to *r* = 3. Each of the two-character elements is repeated 9 times. (**e**) The green lines represent the thumb and index digit trajectories when grasping the sphere along the depth axis. The purple line represents the last 250 mm of the movement path of the midpoint between the fingertip positions. The values above the purple line show the distances from movement end along the path. The projection of the purple line (arrow) on the sagittal axis (dashed line) represents the hand position at a given point of the movement path. The dotted lines connect the thumb and index digit when both were at the same point of the movement path. The length of the dotted lines thus corresponds to the grip aperture.

The visual stimulus was a high-contrast random-dot sphere (radius: 30 mm, see Fig. 6a) presented in stereo. The sphere was simulated at four different distances (420 mm, 450 mm, 480 mm and 510 mm) along the line of sight. A styrofoam sphere (radius: 30 mm, see Fig. 6b) was mounted on a linear positioning stage and its position was adjusted on each trial to match the position of the visual stimulus. Thus, participants always received consistent visual and haptic feedback about the object position and its dimension.

At the beginning of each trial, participants positioned their right hand on a pole which was positioned 270 mm to the right and 150 mm in front of them and which was 300 mm below their line of sight. From this starting position, participants could easily reach behind the mirror to perform reaching and grasping movements. The visual feedback of both the thumb and the index finger was provided by displaying two small virtual spheres which matched the position of the tips of the two digits (see Fig. 6b).

### Procedure

The experiments were performed in a dark room. Participants were seated in front of the slanted mirror with their head positioned on a chin rest. A custon stereo-test was used to assess the stereo vision of the participants. During this test participants were asked to report whether a frontoparallel disparity-defined surface with different degrees of curvature (base-to-peak depth differences between 5 mm and 30 mm) was bended toward or away from them. All participants passed this test. Experimental trials were preceded by a set of practice trials to get accustomed to the task. Each trial started with the thumb and index fingertips in contact and resting on the top of the pole. As soon as the visual stimulus was displayed participants reached towards and grasped with a precision grip the visual sphere that was coincident with the real object. Viewing geometry (i.e., the relation between the viewing direction and the final grasp axis orientation) was manipulated in the two experiments^5,38^. In Experiment 1, participants grasped the sphere along the depth axis by positioning the thumb and index finger on the front and back sides, respectively. In Experiment 2 they grasped the sphere along an oblique axis by positioning the thumb on the bottom left side and the index finger on the top right side (see Fig. 6b). To facilitate the correct positioning of the digits, we displayed a 90 mm long thin line passing through the sphere and aligned with the requested final grasp orientation. The digits became visible shortly after the start of the movement and remained visible until the end of the trial. The trial was terminated once the object was grasped and when both digits stayed for at least 500 ms on the object’s surface. The monitor then turned black and the participants moved their hand back to the starting position. Before the start of the next trial the real object and the monitor moved to the new position. The inter-trial interval was always 7.5 s.

### Stimulus ordering: de Bruijn sequences

To properly address sequential effects, we built fully counterbalanced ordered sets in which every stimulus type (object’s distance) was *equally likely* to be preceded by every other stimulus type including itself. These sets were based on the de Bruijn sequences^13^. A de Bruijn sequence corresponds to an Eulerian cycle on a de Bruijn graph and it has the great advantage that it is the shortest sequence of stimuli that ensures that all possible stimuli combinations of a certain length are presented (see Fig. 6c). The length of each stimulus combination is defined by the order of the de Bruijn sequence. In the current study, we were interested in the effect of the immediately preceding stimulus and we have thus used de Bruijn sequences of order 2. The length of each sequence was equivalent to *k*^*n*^*r*^*n*^ + 1, where *k* is number of different stimuli (in our case, *k* = 4), *n* is the length of each sequential combination (in our case, *n* = 2), and *r* is a repetition factor (in our case, *r* = 3) which allows for multiple repetitions of each sequential combination. Thus, each sequence was 145 trials long and was composed by 9 repetitions of each of the 16 unique two-trials-long stimuli combinations. For each participant a unique trial sequence was created (for an example, see Fig. 6d).

### Data analysis

Raw data were processed and analyzed using R^39^. To smooth and differentiate the positional data we used a 2nd order Savitzky-Golay filter with a window size of 41 points and we then computed velocities and accelerations in three-dimensional space for each fingertip and the wrist. The moment of the lowest, non-repeating wrist velocity value before the continuously increasing wrist velocity values was the point used to define the start of the movement^5^. The end of the movement was defined on the basis of the Multiple Sources of Information method^40^. We used the following criteria: the closer the grip aperture is to the diameter of the sphere the more probable it is that the movement is close to its end, at the same time, grip aperture should be still decreasing, the second derivative of grip aperture should be positive, and the velocities of the wrist, thumb and index finger should be as low as possible. In addition, to capture the first occurrence at which the above criteria were met we decreased over time the probability of a moment being the end of the movement.

In total, 2030 trials were recorded in each experiment. Trials in which the end of the movement was not captured correctly or in which the missing marker samples could not be reconstructed using interpolation were discarded from further analysis. Moreover, the first trial of each trial sequence was discarded, because it was not preceded by any other trial and it was therefore not suitable for the analysis of sequential effects. The total number of trials was thus 1993 and 1878 in Experiment 1 and 2, respectively.

The two main dependent variables on which we have focused our analyses were the grip aperture and the hand position. The grip aperture was computed by taking the Euclidean distance between the fingertips of the thumb and the index finger. The hand position was defined as the relative distance along the sagittal axis between the hand (i.e., the midpoint between the fingertip positions) and the object’s center (see Fig. 6e). A hand position value of 0 mm indicates that the hand was grasping the object at the end of the movement. The more negative the hand position value, the more distant (along the sagittal axis) the hand was from the object.

The grip aperture and the hand position were analyzed along the last 250 mm of the 3D movement path before contact with the object was made (purple line in Fig. 6e). We chose this segment because it covers the whole portion of the movement during which the grip aperture is reaching its maximum peak before closing on the object. Because the number of samples inevitably varies across trials, the trajectories were resampled in 251 points evenly spaced along the three-dimensional trajectory in the range from −250 mm to 0 mm (movement end) in 1 mm steps using cubic spline interpolation.

We used linear mixed-effects models to estimate the current distance effect on the grip aperture, and the previous distance effect on the grip aperture and on the hand position. We determined the optimal structure of the random component using likelihood ratio testing by comparing nested models fitted with restricted estimate maximum likelihood (REML). Models with independent random intercept and random slope terms for participants were the most parsimonious models in both Experiment 1 and Experiment 2. We fitted separate linear mixed-effects models to extract the slope parameters for each point along the movement path to construct the slope profiles. These slope profiles thus represent the progress of the current distance and the previous distance effects along the trajectory. Moreover, we have computed 95% Wald confidence intervals of the slope parameters for each point along the movement path. These confidence intervals were used to determine the strength of the current and previous distance effects.

Linear regression models were used to construct the slope profiles for the current and previous distance effects on grip aperture for each individual participant. Then, for each participant, we have extracted the minimum peak for the current distance effect and the maximum peak for the previous distance effect. These peaks were then used to assess the relationship between current and previous distance effects by computing the slope and intercept of a linear regression model and the correlation coefficient. We assessed the variability of these estimates by computing the 95% confidence intervals with the adjusted bootstrap percentile (BCa) method (10,000 bootstraps).

### Modeling

An object of depth *z* viewed at a fixation distance *d*_*f*_ is subject to systematic perceptual distortions if its shape is only specified by binocular disparities^7–11^. The reason for this bias is attributed to a wrong estimate of the object egocentric distance which yields an inaccurate scaling of retinal disparities. The bias in distance estimate has been shown to be described by the following equation:

5 $${d}_{s}={d}_{0}+k({d}_{f}-{d}_{0}),$$

where *d*_0_ indicates the specific distance where the estimate is veridical. If *d*_*f*_ scales the retinal disparities, then it can be shown that the estimated depth of the object ($\hat{z}$) is related to the true depth *z* through the following equation^6,11^:

6 $$\hat{z}={(\frac{{d}_{s}}{{d}_{f}})}^{2}z={(\frac{{d}_{0}+k({d}_{f}-{d}_{0})}{{d}_{f}})}^{2}z$$

The squared term, which equals 1 for veridical scaling, varies in a nonlinear fashion with the distance of the object *d*_*f*_. However, for a small range of viewing distances this equation can be approximated by a line and we can thus expand it with a Taylor series:

7 $$\hat{z}={\hat{z}({d}_{0})+\frac{\delta \hat{z}}{\delta {d}_{f}}|}_{{d}_{f}={d}_{0}}({d}_{f}-{d}_{0})=z+{s}_{dz}({d}_{f}-{d}_{0}),$$

where *s*_*dz*_ is the slope of the function relating perceived depth to viewing distance, which can be obtained by deriving equation (6) with respect to *d*_*f*_: ${s}_{dz}=2z\tfrac{k-1}{{d}_{0}}$. Since in our experiment the depth of the object corresponds to the diameter of the sphere *D*: ${s}_{dz}=2D\tfrac{k-1}{{d}_{0}}$.

Wrong estimates of fixation distance, which give rise to the lack of depth constancy described by equation (6), can also affect size estimates of segments lying in the frontoparallel plane. If we indicate with $\hat{x}$ the estimated size of a frontoparallel segment of length *x* viewed at a distance *d*_*f*_ then scaling retinal size with the wrong distance *z*_*s*_ will yield a biased 2D size estimate:

8 $$\hat{x}=(\frac{{d}_{s}}{{d}_{f}})x=(\frac{{d}_{0}+k({d}_{f}-{d}_{0})}{{d}_{f}})x$$

Note that the lack of size constancy described by this equation is less severe than the lack of depth constancy, since the latter is governed by a quadratic function of scaling distance (Eq.(6)). Any size estimate of a line segment slanted away from the frontoparallel plane will be subject to the combined effect of size and depth estimates. Specifically, the estimated length of a slanted segment of components *z*_*l*_ and *x*_*l*_ will be given by:

9 $$\hat{l}=\sqrt{{{\hat{x}}_{l}}^{2}+{{\hat{z}}_{l}}^{2}}$$

As we did for the function relating depth estimates to distance also the estimated length can be expressed, in first approximation, as a linear function of distance:

10 $$\hat{l}={\hat{l}({d}_{0})+\frac{\delta \hat{l}}{\delta {d}_{f}}|}_{{d}_{f}={d}_{0}}({d}_{f}-{d}_{0})=l+{s}_{dl}({d}_{f}-{d}_{0}),$$

where $l=\sqrt{{x}_{l}^{2}+{z}_{l}^{2}}$ is the true length of the segment. Since in this study the line segment was the diameter of the sphere: *l* = *D*. If we plug equations (6) and (8) in equation (9) and derive with respect to *d*_*f*_ we obtain: ${s}_{dl}=\frac{3}{2}D\tfrac{k-1}{{d}_{0}}$. Note how the slope *s*_*dl*_ quantifying the lack of size constancy of a slanted segment is $\frac{3}{4}$ the slope *s*_*dz*_ expressing the lack of depth constancy.

In summary, the Euclidean distance estimate of two diametrically opposed points on a sphere of diameter *r* will depend on the distance *d*_*f*_ through an equation:

11 $$\hat{r}=r+{s}_{d}({d}_{f}-{d}_{0}),$$

where the magnitude of *s*_*d*_ depends on the orientation of the segment identified by the two points. A grasping action where the thumb and the index digit aim at these two points can be described by the temporal variation of the grip aperture *ga*(*t*) which is function of the planned grip aperture, *r*_*p*_: *ga*(*t*) = *g*(*r*_*p*_, *t*).

Since we postulate an adaptive visuomotor system, which corrects the visuomotor mapping at each trial on the basis of the detected sensory prediction errors, the planned movement at the (*n* − 1)th grasp depends on both $\hat{r}$ and an internal state *X*_*n*−1_: ${r}_{{p}_{n-1}}={\hat{r}}_{n-1}+{X}_{n-1}$. Given that in our case the object size is always the same, *r* is constant and thus ${\hat{r}}_{n-1}$ depends only on the fixation distance. When the object is grasped, a discrepancy can in theory be detected between the haptic feedback expected on the basis of the planned grip ${r}_{{p}_{n-1}}$ and the felt size *r*:

12 $${\varepsilon }_{{g}_{n-1}}={r}_{{p}_{n-1}}-r={\hat{r}}_{n-1}+{X}_{n-1}-r={s}_{d}({d}_{{f}_{n-1}}-{d}_{0})+{X}_{n-1}.$$

This error leads to a correction in the internal state:

13 $${X}_{n}=a{X}_{n-1}-b{\varepsilon }_{{g}_{n}}=(a-b){X}_{n-1}-b{s}_{d}({d}_{{f}_{n-1}}-{d}_{0}),$$

where *a* is a retention parameter and *b* a correction parameter. Since ${\hat{r}}_{n}=r+{s}_{d}({d}_{{f}_{n}}-{d}_{0})$, the programmed grip for the following trial is now:

14 $${r}_{{p}_{n}}={\hat{r}}_{n}+{X}_{n}=r+{s}_{d}({d}_{{f}_{n}}-{d}_{0})-b{s}_{d}({d}_{{f}_{n-1}}-{d}_{0})+(a-b){X}_{n-1}$$

Therefore, the planned grip for the *n*th trial depends both on the current object distance $({d}_{{f}_{n}})$ and the previous object distance $({d}_{{f}_{n}})$.

We now can assume that the grasping function *g*(*r*_*p*_, *t*) can also be approximated locally at the point of veridicality by a linear function:

15 $$ga(t)\approx {g(r,t)+\frac{\delta g}{\delta {r}_{p}}|}_{{r}_{p}=r}({r}_{p}-r)=g(r,t)+{g}_{{r}_{p}}(t)({r}_{p}-r\mathrm{)}.$$

Therefore, at the trial *n* the grip aperture should be:

16 $$g{a}_{n}(t)={g}_{{r}_{p}}(t)({r}_{{p}_{n}}-z)={g}_{{r}_{p}}(t)\,({s}_{d}({d}_{{f}_{n}}-{d}_{0})-b{s}_{d}({d}_{{f}_{n-1}}-{d}_{0})+(a-b){X}_{n-1}\mathrm{)}.$$

Thus, the grip aperture depends both on the distance of the current trial and on the distance of the previous trial. From the above equation we see that the slope relating grip aperture and the current object distance is:

17 $${{\rm{slope}}}_{n}(t)={g}_{{r}_{p}}(t){s}_{d}$$

and the slope relating grip aperture and the previous object distance is:

18 $${{\rm{slope}}}_{n-1}(t)=-\,b{g}_{{r}_{p}}(t){s}_{d}.$$

## Data Availability

The authors can make the data available upon request.
